# Dr. Stricker’s Manual of Histology

**Published:** 1873-07

**Authors:** D. C. Hawxhurst


### DR. STRICKER'S MANUAL OF HISTOLOGY.

BY D. C. HAWXHURST.

There are some gentlemen in the dental profession (only a few, I believe) who have no patience with any dental literature that does not relate directly to practice. They affect to despise all writing on those sciences which underlie practice. They are like the cock scratching on a dung hill with two or three pullets, who happened to turn up a jewel; he knew what it was well enough by its bright lustre, but he affected a gay contempt for it, and expressed himself in this wise: "Indeed you are very fine, but you have no business here. My taste lies quite another way; and I would rather have a grain of barley than all the jewels under the sun." These gentlemen prefer some grains of information about edging a file or filling a tooth, to all the imperishable jewels of science. And yet it is true that the highest successes in practice are achieved by those most profoundly grounded in this same dispised science.

Perhaps Dr. Stricker's Manual of Histology will never be called to feel the force of such strictures as are thus characterised by the attitude of the cock toward the jewel. I can conceive how its bright attractions may even win such scoffers at science.

No book, since the great work of Kolliker, has appeared that can at all compare with this translation of Dr. Stricker's Manual. It differs from Dr. Kolliker's work as regards size and method of production. Kolliker's work was mainly a result of the labors of one man. This manual combines the labors of more than thirty specialists directly into its pages without any remanipulation.

In such a science as histology great advantage must result from thus associating the labors of special workers in its different departments, for this province of knowledge is too vast to be acquired by a single mind. The specialist focuses the light upon his own spot in science. He studies life in a narrow area, brilliantly illuminated to his own eye, but shaded to the more distant vision of others. He will therefore be likely to talk more intelligently about his own department than any other man not engaged in his specialty, and only knowing it at second hand.

And this seems to have been the view of Dr. Stricker, for he has chosen from a large number of experts, already distinguished for their discoveries, a roll of names great enough to cover the whole field of histology. This able corps of scientific men, whose business it has been to study the structures of animal life, have endeavored to set before the reader what is at present known of the minute constitution of every tissue and fluid of the animal body, and also the character and mode of organization of their ultimate parts.

The richness of results which we here find poured without stint into this large volume, is somewhat appalling to one who has been accustomed to master his text book in the short space of three months. But this is no ordinary text-book. It cannot remain undiscovered that this manual is one of those books from which text books are compiled. It contains the original point of knowledge, fresh from the basket of the discoverer and picker, with the dew still dripping and the bloom still on.

I am sure that the reader will be glad to see the name of

Kuhne over two articles on the Termination of Nerve in Muscle, and, not less so, to see an article by Buch on the Examination of Muscle in Polarized Light.

Pfluger's admirable article on the salivary glands will justly interest the student ; he should remember that Plugger's statements concerning the distribution of nerve fibres to the gland cells have not been confirmed by other observers, and have therefore fallen into discredit.

It should be observed that the structure of the fibres of voluntary muscles has not been explained with sufficient fulness. Noting this omission while treating of some allied topics, Prof. Stricker promises a fuller treatment in the second part. Turning now to the promised article we find a few pages mainly critical, and certainly very inadequate.

It would be extraordinary if a first edition of so large and so solid a work as this did not contain some instances of oversight and error. Even so acute an observer as Rollett must regret that he did not study the views of Rauvier before he wrote his paper on connective cells.

The disciples of Dr. Beale will find Hering's doctrine on the relation of liner cells to the gall capillaries, at variance with their accustomed view, and will be profited by the careful study of the later doctrine.

Meynert has attempted to demonstrate the connection of all parts of the brain with each other, and has expended immense labor in showing or attempting to show what the exact nature of the relation is. He holds that every part of the brain is connected with certain paths of sensation and motion, and endeavors to show the laws of action. His style is obscure and full of ambiguities, and I am glad that I am not the only reader who has found his meaning inextricable. I have hesitated to condemn this paper of Meynert for I well know that the subject is a difficult one. I hope what I have written may not deter new readers from an impartial examination.

The illustrations are generally admirable, I have noticed one on page 382, however, which by some oversight was not lettered.

These are a few blemishes in a work whose merits are much less easily enumerated.

It is difficult to pass over Dr. Stricker's two chapters on the character of cells without the highest commendation. They are as interesting as a fairy story, and are as a whole the best exposition which we now have of the cell.

Nor can too much be said in praise of Max Schultz's exposition of the minute structure of the nervous system.

Ludwig on the kidney, Waldeyer on the teeth, and thirty or forty other papers must all receive praise if one should set for himself the task of commending all that is excellent in this volume. It contains a larger mass of accurate knowledge than has ever before been brought together; with a keener scrutiny of each department, and a mastery of treatment more perfect, than can possibly be found in a text-book produced by a single author. If it is not symmetrical in its treatment of each industrial topic it is because much is yet unknown, and because the authors have chosen to walk a path thickly strewn with facts rather than ascend into the regions of hypothesis and imagination.

Histology is a growing science. In it are at work a host of keen eyes, and shrewd intellects, unearthing new facts in the old departments, and opening up new fields of inquiry, discovering new principles, and developing new methods. The proportions of the science grow visibly, and are likely to leave behind every book that does not run rapidly through a succession of editions and revisions. The best that a book can do is to reflect to the reader the state of knowledge on the subject of which it treats at the time of its publication. The course of discovery being forever onward, new additions are made which the book can tell us nothing about. There is no exception to this rule in case of Dr. Stricker's Manual. The reader and student must note the progress of discovery in the scientific journals, and make corrections for himself.

But making due allowance for all the faults which are to be found in this book, it must be said that it is, and was intended to be, one of the solidist scientific works of the year. It is, indeed, quite refreshing in this age of book-making, in which the most necessary material are good paper and neat binding, well blown up with windy imaginings and plausible hypotheses—it is especially refreshing to meet with a volume so rich in facts and so full of original studies of special subjects.

Nor is this a book that can be mastered without labor. It will call out the student's best powers. Some books are so carefully accurate, so full of gradation and order that they make no demand for thought. It might almost be said that they count the reader a fool, and do the whole work for him, gorging the passive mind with weakly diluted knowledge.

Such is not the character of this manual. By the wide range and exceeding minuteness of its inquiries it calls for intelligent and determined labor in the reader. But still more by the habit which many of its contributors have, of presuming upon the reader's learning and sagacity, it appeals to his best faculties, and is wonderfully suggestive to him who can take a hint from his author, and study it until it expands and ripens into results.

It is fitted to provoke and stimulate the student's faculties to their fullest activity. Ambitious young men, practitioners of either medicine or dentistry, who wish to explore thoroughly those sciences which form the groundwork of the laws of cure, will find no other work in the department of histology so helpful to them, for the simple reason that no other work in English is so exhaustive and complete.

On the night before the celebrated battle of Pharsalia which he believed would decide the destiny of nations, Brutus sat in his tent beneath the stars, reading and making notes with his pen. Amid all the apprehension and preparation which precede a battle, this great mind found time for that communion with literature, which seemed native to it. If the practitioner finds his mind too easily diverted by the demands of business, from ordinary books, let him try Dr. Stricker's Manual. He will find in that an anchorage sufficiently heavy to hold him. Its glimpses of histological physiology, its revelations of morphology, and its several hundred beautiful illustrative engravings, are all attractions. I cannot but hope that they may draw him to his desk during many an otherwise wasted evening hour.

